# (2,2′-Dimethyl-4,4′-bi-1,3-thia­zole-κ^2^ *N*,*N*′)diiodidomercury(II)

**DOI:** 10.1107/S1600536810029302

**Published:** 2010-07-31

**Authors:** Anita Abedi, Effat Yahyazade Bali

**Affiliations:** a Department of Chemistry, Islamic Azad University, North Tehran Branch, Tehran, Iran

## Abstract

In the title compound, [HgI_2_(C_8_H_8_N_2_S_2_)], the Hg^II^ atom is four-coordinated in a distorted tetra­hedral geometry by two N atoms from a 2,2′-dimethyl-4,4′-bithia­zole ligand and two I atoms. In the crystal structure, adjacent mol­ecules are connected by π–π contacts between the thia­zole rings [centroid–centroid distance = 3.591 (3) Å].

## Related literature

For metal complexes with the 2,2′-dimethyl-4,4′-bithia­zole ligand, see: Al-Hashemi *et al.* (2009 ▶); Khavasi *et al.* (2008 ▶); Notash *et al.* (2008 ▶). For related structures, see: Safari *et al.* (2009 ▶); Tadayon Pour *et al.* (2008 ▶); Yousefi *et al.* (2008 ▶).
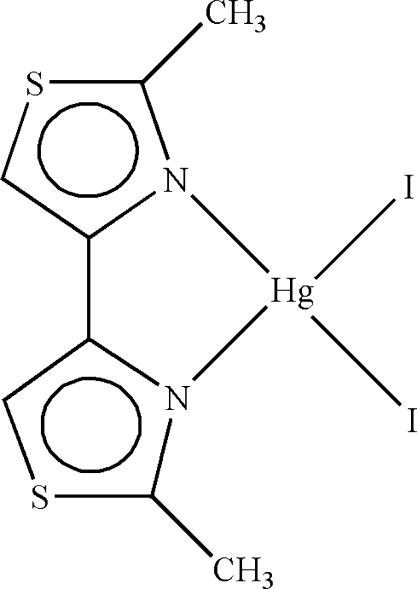


## Experimental

### 

#### Crystal data

[HgI_2_(C_8_H_8_N_2_S_2_)]
*M* *_r_* = 650.67
Orthorhombic, 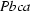

*a* = 12.9059 (10) Å
*b* = 14.8605 (11) Å
*c* = 14.9432 (11) Å
*V* = 2865.9 (4) Å^3^
*Z* = 8
Mo *K*α radiation
μ = 15.31 mm^−1^
*T* = 100 K
0.18 × 0.16 × 0.11 mm

#### Data collection

Bruker APEXII CCD diffractometer
Absorption correction: multi-scan (*SADABS*; Bruker, 2001 ▶) *T*_min_ = 0.085, *T*_max_ = 0.191
27850 measured reflections
3135 independent reflections
2746 reflections with *I* > 2σ(*I*)
*R*_int_ = 0.060

#### Refinement

*R*[*F* ^2^ > 2σ(*F* ^2^)] = 0.025
*wR*(*F* ^2^) = 0.058
*S* = 1.00
3135 reflections
138 parameters
H-atom parameters constrained
Δρ_max_ = 0.74 e Å^−3^
Δρ_min_ = −1.36 e Å^−3^

### 

Data collection: *APEX2* (Bruker, 2007 ▶); cell refinement: *SAINT-Plus* (Bruker, 2007 ▶); data reduction: *SAINT-Plus*; program(s) used to solve structure: *SHELXTL* (Sheldrick, 2008 ▶); program(s) used to refine structure: *SHELXTL*; molecular graphics: *SHELXTL*; software used to prepare material for publication: *SHELXTL*.

## Supplementary Material

Crystal structure: contains datablocks I. DOI: 10.1107/S1600536810029302/hy2334sup1.cif

Structure factors: contains datablocks I. DOI: 10.1107/S1600536810029302/hy2334Isup2.hkl

Additional supplementary materials:  crystallographic information; 3D view; checkCIF report

## Figures and Tables

**Table 1:** Selected bond lengths (Å)

Hg1—N1	2.397 (4)
Hg1—N2	2.408 (4)
Hg1—I1	2.6600 (4)
Hg1—I2	2.6592 (4)

**Fig. 1.**
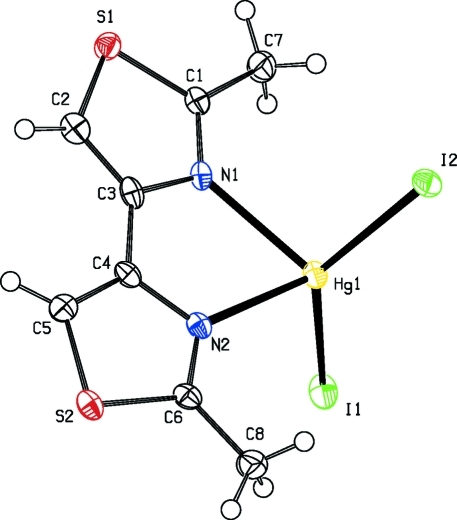
The molecular structure of the title compound. Displacement ellipsoids are drawn at the 50% probability level.

**Fig. 2.**
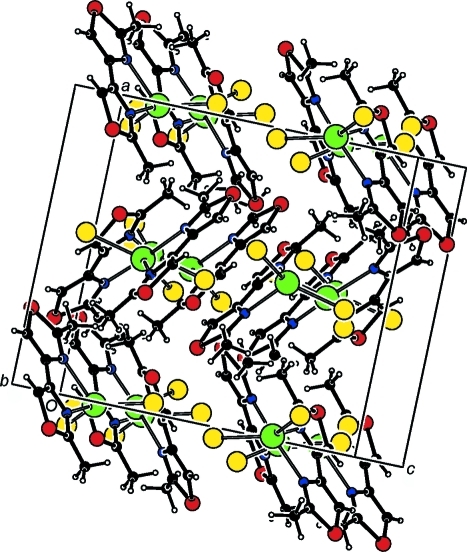
Crystal packing diagram of the title compound.

## supplementary crystallographic information

### Comment

Khavasi *et al.* (2008) reported the synthesis and structure of 2,2'-dimethyl-4,4'-bithiazole (dm4bt) by single crystal X-ray diffraction methods. Dm4bt is a good bidentate ligand, and numerous complexes with dm4bt have been prepared, such as those of zinc (Khavasi *et al.*, 2008), thallium (Notash *et al.*, 2008), cadmium (Notash *et al.*, 2008) and copper (Al-Hashemi *et al.*, 2009). For further investigation of dm4bt, we synthezised the title complex, and report herein its crystal structure.

In the title compound (Fig. 1), the Hg^II^ atom is four-coordinated in a distorted tetrahedral geometry by two N atoms from a 2,2'-dimethyl-4,4'-bithiazole ligand and two I atoms. The Hg—N and Hg—I bond lengths and angles (Table 1) are within normal range of [Hg(SCN)_2_(dm4bt)] (Safari *et al.*, 2009), [HgI_2_(4,4'-dmbpy)] (Yousefi *et al.*, 2008) and [HgI_2_(5,5'-dmbpy)] (Tadayon Pour *et al.*, 2008) (4,4'-dmbpy = 4,4'-dimethyl-2,2'-bipyridine; 5,5'-dmbpy = 5,5'-dimethyl-2, 2'-bipyridine). In the crystal structure, π–π contacts (Fig. 2) between the thiazole rings, *Cg*2···*Cg*3^i^ [symmetry code: (i) 1-x, 1-y, -z. *Cg*2 and *Cg*3 are centroids of the S1, C1, N1, C3, C2 ring and the S2, C5, C4, N2, C6 ring], stabilize the structure, with a centroid–centroid distance of 3.591 (3) Å.

### Experimental

A solution of 2,2'-dimethyl-4,4'-bithiazole (0.20 g, 1.00 mmol) in methanol (15 ml) was added to a solution of HgI_2_ (0.46 g, 1.00 mmol) in methanol (15 ml) at room temperature. Crystals suitable for X-ray diffraction experiment were obtained after one week by methanol diffusion to a colorless solution of the title compound in DMSO (yield: 0.48 g, 73.8%).

### Refinement

H atoms were positioned geometrically and refined as riding atoms, with C—H = 0.95 (CH) and 0.98 (CH_3_) Å and with *U*_iso_(H) = 1.2(1.5 for methyl)*U*_eq_(C).

### Crystal data

**Table:**

[HgI_2_(C_8_H_8_N_2_S_2_)]	*D*_x_ = 3.016 Mg m^−^^3^
*M**_r_* = 650.67	Mo *K*α radiation, λ = 0.71073 Å
Orthorhombic, *P**b**c**a*	Cell parameters from 4823 reflections
*a* = 12.9059 (10) Å	θ = 4–27°
*b* = 14.8605 (11) Å	µ = 15.31 mm^−^^1^
*c* = 14.9432 (11) Å	*T* = 100 K
*V* = 2865.9 (4) Å^3^	Prism, colorless
*Z* = 8	0.18 × 0.16 × 0.11 mm
*F*(000) = 2304	

### Data collection

**Table:**

Bruker APEXII CCD diffractometer	3135 independent reflections
Radiation source: fine-focus sealed tube	2746 reflections with *I* > 2σ(*I*)
graphite	*R*_int_ = 0.060
φ and ω scans	θ_max_ = 27.0°, θ_min_ = 2.5°
Absorption correction: multi-scan (*SADABS*; Bruker, 2001)	*h* = −16→16
*T*_min_ = 0.085, *T*_max_ = 0.191	*k* = −18→18
27850 measured reflections	*l* = −19→19

### Refinement

**Table:**

Refinement on *F*^2^	Primary atom site location: structure-invariant direct methods
Least-squares matrix: full	Secondary atom site location: difference Fourier map
*R*[*F*^2^ > 2σ(*F*^2^)] = 0.025	Hydrogen site location: inferred from neighbouring sites
*wR*(*F*^2^) = 0.058	H-atom parameters constrained
*S* = 1.00	*w* = 1/[σ^2^(*F*_o_^2^) + (0.030*P*)^2^ + 3.*P*] where *P* = (*F*_o_^2^ + 2*F*_c_^2^)/3
3135 reflections	(Δ/σ)_max_ = 0.002
138 parameters	Δρ_max_ = 0.74 e Å^−^^3^
0 restraints	Δρ_min_ = −1.36 e Å^−^^3^

### Fractional atomic coordinates and isotropic or equivalent isotropic displacement parameters (Å^2^)

**Table:**

	*x*	*y*	*z*	*U*_iso_*/*U*_eq_	
Hg1	0.510845 (16)	0.378820 (14)	0.225249 (14)	0.01739 (7)	
I1	0.59477 (3)	0.23923 (3)	0.14062 (3)	0.02478 (10)	
I2	0.46410 (3)	0.41700 (2)	0.39435 (2)	0.02213 (9)	
S1	0.61688 (11)	0.68779 (9)	0.15627 (10)	0.0197 (3)	
S2	0.24214 (11)	0.45250 (10)	0.00224 (9)	0.0209 (3)	
N1	0.5553 (3)	0.5267 (3)	0.1740 (3)	0.0169 (9)	
N2	0.3846 (3)	0.4293 (3)	0.1177 (3)	0.0158 (9)	
C1	0.6287 (4)	0.5813 (4)	0.2004 (4)	0.0177 (11)	
C2	0.5076 (4)	0.6562 (4)	0.1008 (4)	0.0203 (12)	
H2A	0.4678	0.6948	0.0637	0.024*	
C3	0.4849 (4)	0.5682 (4)	0.1169 (4)	0.0181 (11)	
C4	0.3987 (4)	0.5150 (4)	0.0821 (3)	0.0166 (11)	
C5	0.3287 (4)	0.5378 (4)	0.0189 (4)	0.0186 (11)	
H5A	0.3278	0.5936	−0.0121	0.022*	
C6	0.3056 (4)	0.3887 (4)	0.0811 (4)	0.0177 (11)	
C7	0.7144 (5)	0.5550 (4)	0.2603 (4)	0.0271 (13)	
H7A	0.6860	0.5350	0.3178	0.041*	
H7B	0.7602	0.6066	0.2700	0.041*	
H7C	0.7537	0.5057	0.2330	0.041*	
C8	0.2714 (5)	0.2959 (4)	0.1047 (4)	0.0260 (13)	
H8A	0.2584	0.2923	0.1692	0.039*	
H8B	0.3257	0.2528	0.0882	0.039*	
H8C	0.2076	0.2815	0.0721	0.039*	

### Atomic displacement parameters (Å^2^)

**Table:**

	*U*^11^	*U*^22^	*U*^33^	*U*^12^	*U*^13^	*U*^23^
Hg1	0.01885 (11)	0.01823 (11)	0.01509 (11)	0.00215 (8)	−0.00002 (8)	0.00063 (8)
I1	0.0298 (2)	0.0248 (2)	0.01979 (19)	0.01150 (16)	0.00339 (16)	0.00036 (15)
I2	0.0271 (2)	0.0226 (2)	0.01672 (18)	0.00144 (15)	0.00188 (14)	−0.00428 (14)
S1	0.0204 (7)	0.0173 (7)	0.0214 (7)	0.0005 (5)	−0.0012 (5)	0.0021 (5)
S2	0.0201 (7)	0.0259 (8)	0.0166 (7)	0.0026 (5)	−0.0042 (5)	0.0006 (6)
N1	0.018 (2)	0.019 (2)	0.013 (2)	0.0015 (18)	0.0012 (18)	0.0033 (18)
N2	0.016 (2)	0.018 (2)	0.013 (2)	0.0047 (17)	0.0009 (17)	−0.0022 (18)
C1	0.018 (3)	0.021 (3)	0.014 (3)	0.002 (2)	0.000 (2)	0.000 (2)
C2	0.015 (3)	0.025 (3)	0.021 (3)	0.005 (2)	0.000 (2)	−0.001 (2)
C3	0.017 (3)	0.026 (3)	0.011 (3)	0.005 (2)	0.004 (2)	0.001 (2)
C4	0.015 (3)	0.022 (3)	0.013 (3)	0.002 (2)	0.001 (2)	−0.003 (2)
C5	0.020 (3)	0.018 (3)	0.018 (3)	0.002 (2)	0.007 (2)	−0.003 (2)
C6	0.018 (3)	0.019 (3)	0.016 (3)	0.004 (2)	0.001 (2)	−0.005 (2)
C7	0.027 (3)	0.027 (3)	0.028 (3)	−0.004 (2)	−0.010 (3)	0.006 (3)
C8	0.028 (3)	0.023 (3)	0.027 (3)	−0.001 (2)	−0.007 (3)	0.000 (3)

### Geometric parameters (Å, °)

**Table:**

Hg1—N1	2.397 (4)	C2—C3	1.361 (8)
Hg1—N2	2.408 (4)	C2—H2A	0.9500
Hg1—I1	2.6600 (4)	C3—C4	1.462 (8)
Hg1—I2	2.6592 (4)	C4—C5	1.349 (7)
S1—C2	1.701 (6)	C5—H5A	0.9500
S1—C1	1.721 (6)	C6—C8	1.491 (8)
S2—C5	1.708 (6)	C7—H7A	0.9800
S2—C6	1.720 (6)	C7—H7B	0.9800
N1—C1	1.309 (7)	C7—H7C	0.9800
N1—C3	1.390 (7)	C8—H8A	0.9800
N2—C6	1.304 (7)	C8—H8B	0.9800
N2—C4	1.393 (7)	C8—H8C	0.9800
C1—C7	1.475 (8)		
			
N1—Hg1—N2	70.32 (15)	N1—C3—C4	118.4 (5)
N1—Hg1—I2	99.35 (11)	C5—C4—N2	114.2 (5)
N2—Hg1—I2	114.46 (10)	C5—C4—C3	128.5 (5)
N1—Hg1—I1	117.74 (11)	N2—C4—C3	117.3 (5)
N2—Hg1—I1	101.61 (10)	C4—C5—S2	110.7 (4)
I2—Hg1—I1	135.280 (14)	C4—C5—H5A	124.7
C2—S1—C1	90.4 (3)	S2—C5—H5A	124.7
C5—S2—C6	89.9 (3)	N2—C6—C8	124.1 (5)
C1—N1—C3	112.5 (5)	N2—C6—S2	113.9 (4)
C1—N1—Hg1	130.2 (4)	C8—C6—S2	122.1 (4)
C3—N1—Hg1	116.5 (3)	C1—C7—H7A	109.5
C6—N2—C4	111.4 (4)	C1—C7—H7B	109.5
C6—N2—Hg1	131.7 (4)	H7A—C7—H7B	109.5
C4—N2—Hg1	116.9 (3)	C1—C7—H7C	109.5
N1—C1—C7	124.1 (5)	H7A—C7—H7C	109.5
N1—C1—S1	113.0 (4)	H7B—C7—H7C	109.5
C7—C1—S1	122.9 (4)	C6—C8—H8A	109.5
C3—C2—S1	110.9 (4)	C6—C8—H8B	109.5
C3—C2—H2A	124.5	H8A—C8—H8B	109.5
S1—C2—H2A	124.5	C6—C8—H8C	109.5
C2—C3—N1	113.2 (5)	H8A—C8—H8C	109.5
C2—C3—C4	128.4 (5)	H8B—C8—H8C	109.5
			
N2—Hg1—N1—C1	174.2 (5)	C1—N1—C3—C2	0.1 (7)
I2—Hg1—N1—C1	61.5 (5)	Hg1—N1—C3—C2	171.1 (4)
I1—Hg1—N1—C1	−92.7 (5)	C1—N1—C3—C4	180.0 (5)
N2—Hg1—N1—C3	5.1 (3)	Hg1—N1—C3—C4	−9.0 (6)
I2—Hg1—N1—C3	−107.6 (4)	C6—N2—C4—C5	0.4 (6)
I1—Hg1—N1—C3	98.2 (4)	Hg1—N2—C4—C5	176.8 (4)
N1—Hg1—N2—C6	174.7 (5)	C6—N2—C4—C3	−179.8 (5)
I2—Hg1—N2—C6	−93.8 (5)	Hg1—N2—C4—C3	−3.5 (6)
I1—Hg1—N2—C6	59.1 (5)	C2—C3—C4—C5	8.0 (9)
N1—Hg1—N2—C4	−0.7 (3)	N1—C3—C4—C5	−171.9 (5)
I2—Hg1—N2—C4	90.8 (3)	C2—C3—C4—N2	−171.8 (5)
I1—Hg1—N2—C4	−116.3 (3)	N1—C3—C4—N2	8.4 (7)
C3—N1—C1—C7	−179.2 (5)	N2—C4—C5—S2	0.3 (6)
Hg1—N1—C1—C7	11.3 (8)	C3—C4—C5—S2	−179.5 (4)
C3—N1—C1—S1	−0.4 (6)	C6—S2—C5—C4	−0.6 (4)
Hg1—N1—C1—S1	−169.9 (2)	C4—N2—C6—C8	179.5 (5)
C2—S1—C1—N1	0.4 (4)	Hg1—N2—C6—C8	3.9 (8)
C2—S1—C1—C7	179.3 (5)	C4—N2—C6—S2	−0.9 (6)
C1—S1—C2—C3	−0.4 (4)	Hg1—N2—C6—S2	−176.6 (2)
S1—C2—C3—N1	0.2 (6)	C5—S2—C6—N2	0.9 (4)
S1—C2—C3—C4	−179.6 (4)	C5—S2—C6—C8	−179.5 (5)
